# Effect of jujube powder addition on the aroma profile of quinoa snacks (QS)

**DOI:** 10.1002/fsn3.4128

**Published:** 2024-05-06

**Authors:** Jianxin Song, Jiayi Liu, Kaile Wang, Lei Gao, Xiaodong Wang, Jian Peng, Ning Wang

**Affiliations:** ^1^ School of Functional Food and Wine Shenyang Pharmaceutical University Shenyang China; ^2^ School of Biological Science and Food Engineering Chuzhou University Chuzhou China; ^3^ Guangdong Academy of Agricultural Sciences, Guangdong Key Laboratory of Agricultural Products Processing, Key Laboratory of Functional Foods, Ministry of Agriculture and Rural Affairs Sericultural and Agri‐Food Research Institute Guangzhou China; ^4^ School of Light Industry Liaoning University Shenyang China

**Keywords:** aroma profile, e‐nose, GC–MS, jujube powder, quinoa

## Abstract

Quinoa is a full‐nutrition food; however, its poor flavor and small size make it not the best food option for direct consumption. In this study, a quinoa snack (QS, a cake) was developed, and the aroma profile of the products was improved by adding jujube fruit powder (made from dried jujube fruits, from 5% to 30%). Gas chromatography mass spectrum (GC–MS) combined with electronic nose (e‐nose) was applied for characterizing the aroma profiles of QS samples. Results showed a total of 26 aroma compounds were identified in QS samples by GC–MS, and 3‐methylbutanol (from 1525 μg/kg in QS‐30 to 3487 μg/kg in QS‐0), ethanol (from 1126 μg/kg in QS‐0 to 3581 μg/kg in QS‐30), hexanal (from 125.6 μg/kg in QS‐30 to 984.1 μg/kg in QS‐0), and acetaldehyde (from 531.9 μg/kg in QS‐30 to 191.1 μg/kg in QS‐0) were common. The e‐nose response of W1S (sensitive to methane, from 17.50 of QS‐0 to 93.85 of QS‐30) and W1W (sensitive to sulfur‐organic compounds of e‐nose, from 15.57 of QS‐0 to 39.50 of QS‐30) were significantly higher, and significant differences were presented among QS samples. In conclusion, the aroma profile of the QS sample was significantly (*p* < .05) enhanced by the addition of jujube powder, and QS‐30 with the highest jujube content (30%) presented the strongest aroma profile. Moreover, QS samples with different additions of jujube powders could be well distinguished by principal component analysis (PCA), and the combination of e‐nose and GC–MS was effective in the volatile profile analysis of QS samples.

## Graphical abstract

3‐Methylbutanol, ethanol, hexanal, and 2‐methylpropanol were common in quinoa snack (QS) samples. The aroma profile of QS sample was significantly enhanced by the addition of jujube powder. QS‐30 with the highest jujube content (30%) presented the strongest aroma profile. QS samples with different additions of jujube powders could be well distinguished by PCA.

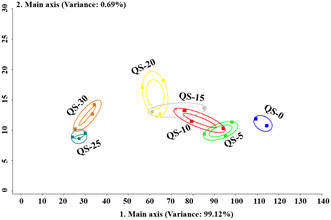

## 1 INTRODUCTION

Quinoa (*Chenopodium quinoa* Willd) is a plant species belonging to the Chenopodiaceae family. As a pseudo‐cereal, quinoa is native to the Andes region, which has a 7000‐year agricultural history (Pearsall, 1992; Song & Tang, 2023). Quinoa has the ability to resist extreme ecological pressures such as high altitude, significant temperature fluctuations, humidity varieties, and pH changes in the soil (Castro et al., 2019; Song, Shao, et al., 2021; Song, Yan, et al., 2021). Quinoa has a balanced amino acid profile, and it is also rich in carbohydrates, protein, and dietary and many functional compositions such as polyphenols, vitamins, saponins, and flavonoids (Chaudhary et al., 2023; Ren et al., 2023). The functional effects of antioxidant, anti‐inflammatory, antidiabetic and antimicrobial activities of quinoa were excellent (Navruz‐Varli & Şanlier, 2016; Shahidi & Chandrasekara, 2013; Starzyńska‐Janiszewska et al., 2023). Hence, quinoa has been recognized as a full‐nutrition food that can provide all the nutrients for the human body by the Food and Agriculture Organization (FAO) (Niu et al., 2023). In daily life, quinoa is commonly processed as salad, bread, biscuits, and cakes (Sezgin & Sanlier, 2019). However, due to its small size, which is covered with chaff, and the light smell and bitter taste caused by saponin, quinoa seed is not the best food option for direct consumption (Cao et al., 2023). Appropriate auxiliary materials need to be added to enhance the flavor quality of quinoa products.

Jujube (*Ziziphus jujuba* Mill.), also known as the red or Chinese date, is a favorite fruit with a unique flavor. In daily life, about 90% of jujubes are consumed in dried form (Song et al., 2020). Jujube powder is one of the major products of dried jujubes (Addo et al., 2019). The dried jujube presents a strong and unique flavor. Volatile compositions such as alcohols, aldehydes, acids, esters, and ketones are abundant in dried jujubes, especially for acids (such as hexanoic acid and octanoic acid) with a sour cheese or fatty smell that make a major contribution to the jujube flavor profile (Song et al., 2020, 2022). Hence, jujube powder is mostly used as an added ingredient in various foods such as bread, milk, and tea (Chen et al., 2014; Liu & Zhao, 2009).

Snacks, such as small packaged cakes, are a series of ready‐to‐eat foods that can meet the demands of flavor, nutrients, and food enjoyment for consumers. Hence, flavor is a key factor that determines the quality and acceptance of snack products. The aim of this study was to developed a baked snack (quinoa snack, QS) mainly made from quinoa. The jujube powder was added to enhance the flavor quality of QS, and the optimum jujube powder content (5%–30%) was investigated based on the aroma profile of QS as well.

## 2 MATERIALS AND METHODS

### 2.1 Material

A total of 5.0 kg of white quinoas (cultivated in Zhuluke Town, Chaoyang, Liaoning Province of China) produced by a process factory (Huaizhi Grain Co., Ltd.) were purchased. All quinoa samples were vacuum packed and stored at 4°C. Inulin (1.0 kg) produced by Henan Wan Bang Industrial Co., Ltd. was obtained. Jujubes of the Huizao variety (5.0 kg) cultivated in Ruoqiang Town, Xinjiang Province, China, were collected. Strong wheat flour (5.0 kg) produced by Wudeli Flour Group with the standard Q/WDL00165 was used. Instant dry yeast (5 g*10) from Angel was applied for fermentation.

### 2.2 Sample preparation

#### 2.2.1 Jujube powder preparation

The jujube fruits were cleaned with running water, and the jujube seeds were removed by a special seed remover (304 stainless steel, KACHEEG, Germany). Then the fruit was cut into 5.0 mm slices and treated with hot‐air drying equipment (DHG‐9123, Jing Hong Laboratory Instruments Co., Ltd., Shanghai, China) at 60°C for 6.0 h. The dried jujube samples were pulverized (JYL‐CO20, Joyoung, Jinan, China), and the powders were passed through an 80‐mesh/inch sieve (Song et al., 2020).

#### 2.2.2 Quinoa flour preparation

About 500 g of quinoa was soaked in 1.5 L of drinking water for 5 h. Then the quinoa sample was manually scrubbed for saponin removal. After being drip‐dried, the quinoa sample was dried at 60°C for 6 h, and then milled and passed through an 80‐mesh/inch sieve.

#### 2.2.3 QS preparation

According to our previous optimizing experiments, the ratio of quinoa to strong wheat flour was 1:1, the content of inulin was 6%, and the instant dry yeast was added with a 5 g/1000 g sample according to the instruction. The variables of jujube powder were 0% (QS‐0), 5% (QS‐5), 10% (QS‐10), 15% (QS‐15), 20% (QS‐20), 25% (QS‐25), and 30% (QS‐30), respectively. For each batch of QS product, the weighted quinoa powder, strong flour, inulin, instant dry yeast, and jujube powder (from 0% to 30%) were well‐mixed in a stainless pipe basin (26 cm, Weiai, Jiangsu, China) with drinking water and covered with cling film, and the sample was fermented for 30 min at room temperature. Then the sample was put into the mold and baked at 150°C for 15 min by an air‐fryer (KL60‐VF506, Joyoung, Jinan, China). After chilling down, the quinoa snack was acquired, as shown in Table 1.

**TABLE 1:** Color and picture of QS with different additions of jujube powder (0–30%).

Sample	Color			Picture
	L*	a*	b*	
QS‐0	49.18 ± 1.33f	10.52 ± 0.36a	64.28 ± 2.31d	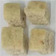
QS‐5	46.22 ± 2.07e	11.37 ± 0.29b	22.76 ± 0.44a	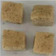
QS‐10	44.35 ± 1.02b	11.38 ± 0.18b	22.40 ± 0.29a	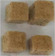
QS‐15	42.15 ± 0.96ab	13.47 ± 0.52c	24.13 ± 0.37b	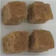
QS‐20	41.63 ± 1.25ab	13.89 ± 0.33c	25.81 ± 0.40bc	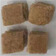
QS‐25	40.28 ± 0.76a	14.03 ± 0.14c	26.19 ± 0.39c	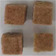
QS‐30	39.14 ± 0.83a	14.11 ± 0.20c	26.53 ± 0.35c	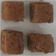

*Note*: QS‐0, QS‐5, QS‐10, QS‐15, QS‐20, QS‐25, and QS‐30 was quinoa snack with the addition of jujube powder of 0%, 5%, 10%, 15%, 20%, 25%, and 30%, respectively. Data are represented as the mean ± SD (standard deviation). Mean values with different lower case letters in the same column correspond to significant differences at *p* < .05.

### 2.3 Color analysis

The color values (L*, a*, b*) of QS samples were analyzed by an automatic calibration color analyzer (WR‐18, Shenzhen Wave Optoelectronics Technology Co., Ltd., China). The lens was gently adhered to the surface of the QS samples, and all the measurements were carried out at room temperature for five repetitions, where L*, a*, and b* represent lightness, redness, and yellowness values, respectively (Sun, Qiao, et al., 2023; Sun, Yu, et al., 2023). The QS samples were arranged in the culture dish and photographed to obtain the physical images.

### 2.4 E‐nose analysis

Aroma profiles of QS samples with different jujube powder additions were analyzed by a PEN 3.5 e‐nose equipped with 10 metal‐oxide semiconductors (Airsense Analytics, GmBH, Schwerin, Germany). According to the method of Song et al. (2022), accurate 5.0 g QS samples were weighed and placed in 20 mL vials for headspace analysis. Briefly, the samples were balanced for 20 min, and G/G0 represents the change in each sensor. The sensor cleaning time was 180 s, and the automatic zero adjustment time was 10 s. The detection time was 120 s with three repetitions. The flow rate of internal and inlet was 600 mL/min.

### 2.5 Volatile compound analysis

A 4000 GC–MS (Varian Inc., Walnut Creek, CA, USA) equipped with a VF‐5 ms capillary column (Agilent Technologies Inc., Santa Clara, CA, USA) was used for the volatile composition analysis of the QS samples. The analysis step was according to the method of Chen, Hu, et al. (2019); Chen, Song, et al. (2019), with some modifications. Briefly, an accurate 5.0 g QS sample was cut into pieces and placed in a 20‐mL solid‐phase micro‐extraction (SPME) vial, which was then sealed with a PTFE‐silicon stopper for bath at 60°C for 30 min. The initial temperature of the oven was 40°C and increased to 150°C at a rate of 4°C/min t, then to 250°C at a rate of 8°C/min for 6 min. The injector temperature and volume were 250°C and 1 μL, respectively. The gas flow rate (99.9% helium) was 1 μL/min with the injection mode of 1/10 split. The scanning range was set at 35–500 m/z to acquire the mass spectra, and the solvent delay was 90 s. The temperature of the ion source and transfer line was 200 and 220°C, respectively. The NIST mass spectral search program v.11.0 (chemdata.nist.gov) was applied for the data processing. Aromatic compounds with a matching degree of over 85 percent were selected for future analysis (Chen, Hu, et al., 2019; Chen, Song, et al., 2019). Hexanal was used as an external standard for the quantitative determination, and the aroma content of the QS sample was calculated by the GC peak areas related to those of hexanal. The standard curve of hexanal was y=0.0001x−0.819, which was established within the concentration range of 0.1 μg/L–10 mg/L (*R* ^2^ = .994).

### 2.6 Statistical analysis

The SPSS software (25.0 version, Inc., Chicago, IL) was applied for the date's treatment of different QS samples. The result was presented as mean ± SD with Duncan's multiple tests at the level of *p* < .05. The differences in characteristic aroma profiles of QS samples were analyzed by principal component analysis (PCA) based on *WinMuster* software.

## 3 RESULTS AND DISCUSSION

### 3.1 Color and appearance of QS samples

Color is one of the most important factors that determines the sensory quality and consumer acceptance of foods (Xing et al., 2022). As shown in Table 1, the color value and figure of QS samples were significantly (*p* < .05) affected by the addition of jujube powders, and a similar conclusion was also reported by Xia et al. (2023) that the product color was significantly affected by jujube kernel powder addition. The color values of L* (ranging from 0 for black to 100 for white), a* (ranging from negative green to positive to red), and b* (ranging from negative blue to positive yellow) of QS samples with different jujube powder additions are shown in Table 1. The range of L* values of QS samples was 39.14 (QS‐30)–49.18 (QS‐0), and the L* values of QS‐0 (49.18) was significantly higher (*p* < .05) than that of QS‐5 (46.22)–QS‐30 (39.24). It indicated that the color of the QS sample became darker with the increasing addition of jujube powders because of the brown color of the jujube powder. The scale of a* value of QS samples was 10.52 (QS‐0)–14.11 (QS‐30), and the supplementation of jujube powders resulted in increase in the redness of QS samples. However, the highest b* value was presented in QS‐0 (64.28) and it significantly (*p* < .05) reduced with the addition of jujube powders (from 22.76 in QS‐5 to 26.53 in QS‐30). In general, the color characteristics of QS samples were determined by their appearances in pictures, as shown in Table 1, and the result was well in agreement with the L*, a*, and b* value analyses.

### 3.2 E‐nose analysis

#### 3.2.1 E‐nose response analysis

**FIGURE 1:**
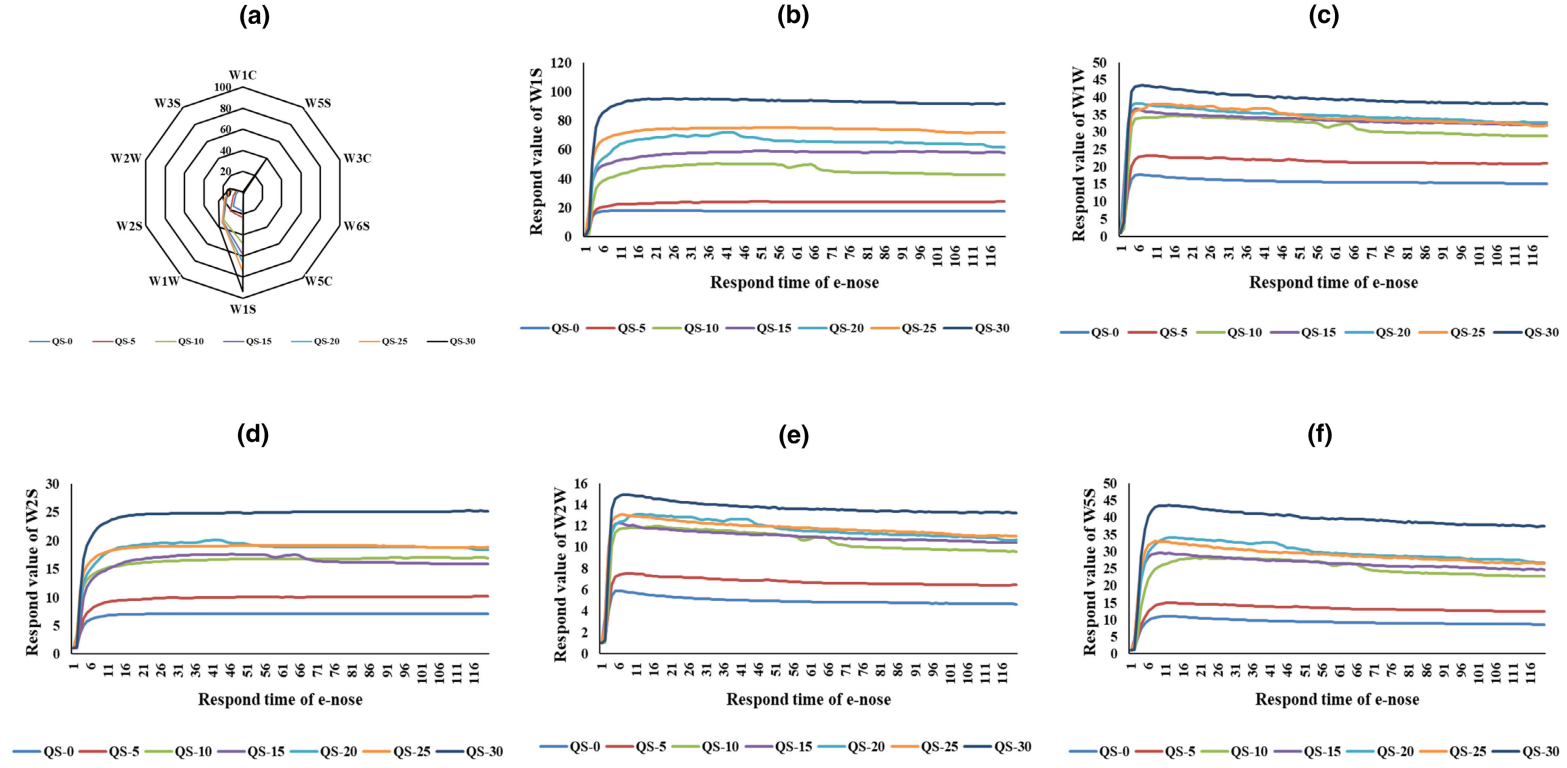
(a) Radar chart of the e‐nose's sensors to quinoa snack (QS) samples. (b) e‐nose response sensor of W1S to QS samples. (c) e‐nose response sensor of W1W to QS samples. (d) e‐nose response sensor of W2S to QS samples. (e) e‐nose response sensor of W2W to QS samples. (f) e‐nose response sensor of W5S to QS samples. Each test of e‐nose was repeated 5 times.

The response of 10 sensors on the e‐nose to QS samples (data in the 100th second was treated) was presented as a radar chart in Figure 1a, where the response was calculated by *G*/*G*_0_ (*G* and *G*_0_ were sensor responses of sample gas and zero gas, respectively; Song et al., 2020). Figure 1a showed the response values of W1S (sensitive to methane), W1W (sensitive to sulfur‐organic compounds), W2S (sensitive to alcohols), W2W (sensitive to sulf‐chlor compounds), and W5S (broad sensitivity) of e‐nose were significant, while other sensor values with little response were less than one (Chen, Hu, et al., 2019; Chen, Song, et al., 2019; Li et al., 2017). The response values of W1S (from 17.50 of QS‐0 to 93.85 of QS‐30) and W1W (from 15.57 of QS‐0 to 39.50 of QS‐30) were significantly higher (*p* < .05), followed by W5S (from 9.27 of QS‐0 to 39.67 of QS‐30), W2S (from 7.06 of QS‐0 to 24.94 of QS‐30), and W2W (from 4.87 of QS‐0 to 13.60 of QS‐30), indicating the aroma profiles of QS samples were significantly different and well analyzed by e‐nose.

In order to make clear the aroma profiles of different QS samples, the comparison of responses of W1S, W1W, W2S, W2W, and W5S was, respectively, presented in Figure 1b–f. Results showed that the sensor response curves of W1S, W1W, W5S, W2S, and W2W of QS samples changed with time and the trends of the sensors were similar. Clearly, the highest response values of W1S, W1W, W5S, W2S, and W2W were, respectively, presented in QS‐30, followed by QS‐25 and QS‐20. Meanwhile, the lowest response values of W1S, W1W, W5S, W2S and W2W was respectively presented in QS‐0. It indicated QS‐30 contained the strongest aroma profile, which was significantly enhanced by jujube powder addition (Singh & Gaur, 2023). However, it was difficult to classify different QS samples by just observing the sensor signals; further PCA was still needed.

#### 3.2.2 PCA classification

**FIGURE 2:**
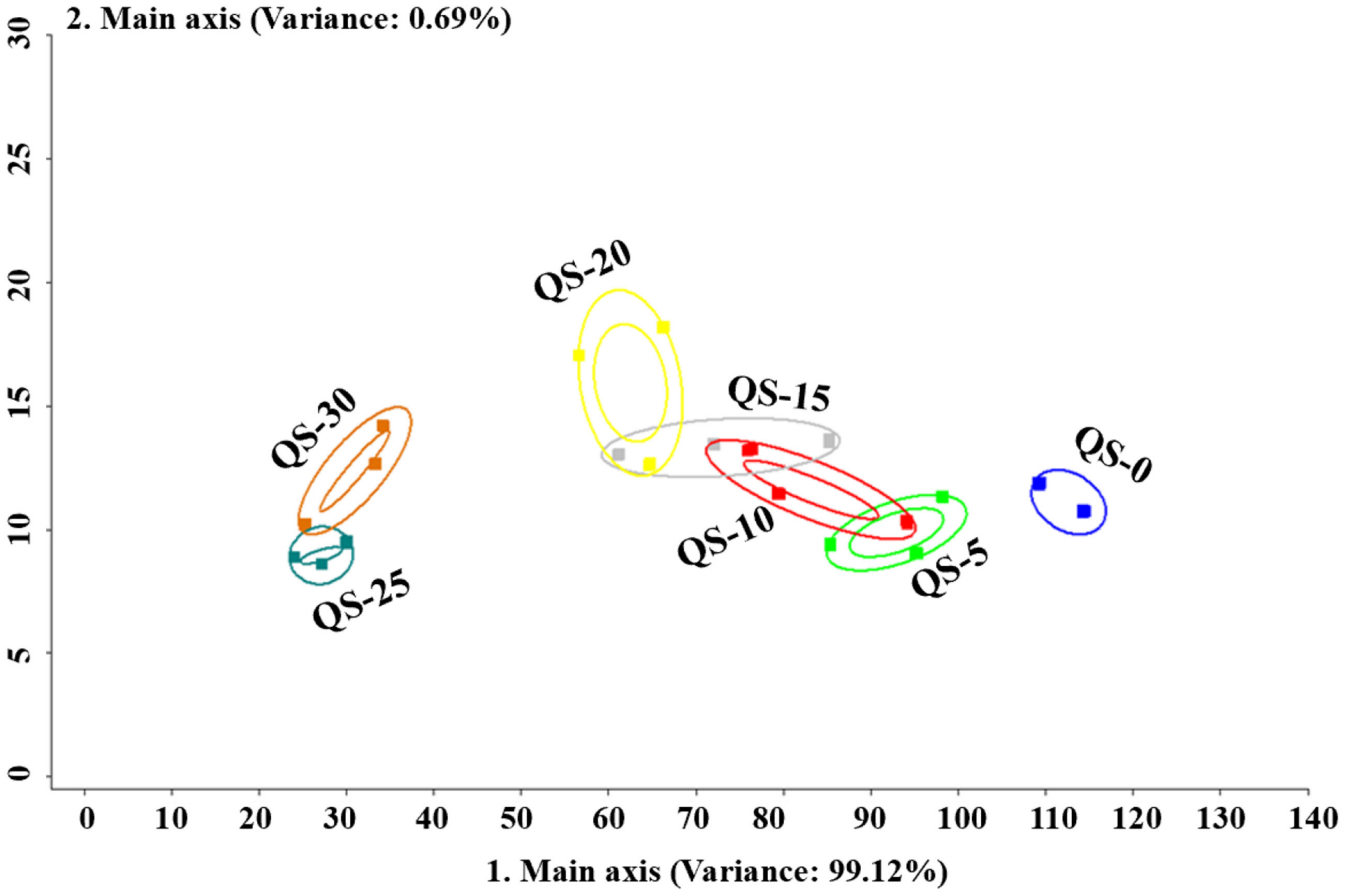
Principal component analysis (PCA) of quinoa snack (QS) samples based on the data from e‐nose.

Principal component analysis (PCA) has the ability to determine complex and difficult‐to‐find variables and evaluate the differences among the samples (Li et al., 2019). In previous studies, aroma profiles of microwave‐dried perilla leaves (Jin et al., 2023), red‐cooked chickens (Sun, Qiao, et al., 2023; Sun, Yu, et al., 2023), and kiwifruits experiencing soft rot (Wang et al., 2023) were successfully characterized by PCA. Hence, PCA was applied for the analysis of QS samples with different jujube powder additions based on the data from e‐nose. As shown in Figure 2, visualization of QS samples with different jujube powder additions was established by PCA, and the total of the first two PCs (main axis 1 of 99.12% plus main axis 2 of 0.69%) of PCA was 99.81%, which was sufficient enough to explain the dataset (Castura et al., 2022). In the PCA plot (Figure 2), QS samples with similar aroma profiles were located overlapping or close to each other, otherwise dissimilar (Song et al., 2020). The sample of QS‐0 was well separated from other samples, indicating the aroma profiles between QS‐0 and QS samples with jujube powder additions were significantly different. QS‐5 was close to QS‐10 and far away from QS‐15, QS‐20, QS‐25, and QS‐30, respectively. QS‐30 was close to QS‐25 and, respectively, far away from other QS samples (Figure 2). It indicated that the aroma profiles of QS samples within a certain range of jujube powder addition were similar, otherwise dissimilar. Generally, the PCA result of the e‐nose can properly distinguish the characteristic aroma profiles of QS samples.

### 3.3 GC–MS analysis

#### 3.3.1 Aroma compositions of QS samples

As shown in Table 2, a total of 26 aroma compounds were identified in QS samples by GC–MS. The amount of aroma compounds in QS‐0 and QS samples (QS‐5, QS‐10, QS‐15, QS‐20, QS‐25, and QS‐30) was 14 and 26, respectively, and the content of the aroma compounds was significantly increased with the increasing addition of jujube powder (from 5% to 30%). It indicated that the additive amount of jujube powder can enrich the aroma profile of the QS sample. Considering the individual aroma compounds in QS samples, 3‐methylbutanol (from 1525 μg/kg in QS‐30 to 3487 μg/kg in QS‐0), ethanol (from 1126 μg/kg in QS‐0 to 3581 μg/kg in QS‐30), hexanal (from 125.6 μg/kg in QS‐30 to 984.1 μg/kg in QS‐0), 2‐methylpropanol (from 251.4 μg/kg in QS‐30 to 774.3 μg/kg in QS‐0), 2,3‐butanedione (from 154.6 μg/kg in QS‐30 to 280.4 μg/kg in QS‐0), methylbutanal (from 174.4 μg/kg in QS‐0 to 1212 μg/kg in QS‐30), acetaldehyde (from 191.1 μg/kg in QS‐0 to 531.9 μg/kg in QS‐30) and acetoin (from 170.3 μg/kg in QS‐0 to 1099 μg/kg in QS‐30) were common in QS samples (Table 2). 2‐Methylpropanol, 2‐methylbutanol, 2,3‐butanedione, and hexanal were major in quinoa (Song, Shao, et al., 2021; Song, Yan, et al., 2021; Yang et al., 2021) and showed a declining trend with the addition of jujube powder because of its higher content in quinoa than in jujube (Gou et al., 2023; Song et al., 2022). Ethanol was also reported in quinoa (Song, Shao, et al., 2021; Song, Yan, et al., 2021), and it was significantly high in QS samples and showed increasing tendency with the addition of jujube powder, because ethanol was formed during the fermentation process (Garrido‐Galand et al., 2021) and jujube powder provided more reducing sugars (Fu et al., 2021). Table 2 showed acetaldehyde, methylbutanal, acetoin, ethyl acetate, and ethyl octanoate presented an increasing tendency in QS samples with the addition of jujube powder. Moreover, aroma compounds such as acetic acid (from 7.86 μg/kg in QS‐5 to 70.34 μg/kg in QS‐30), hexanoic acid (from 20.07 μg/kg in QS‐5 to 96.89 μg/kg in QS‐30), benzaldehyde (from 88.05 μg/kg in QS‐5 to 627.2 μg/kg in QS‐30), and furfural (from 13.64 μg/kg in QS‐5 to 137.9 μg/kg in QS‐30) were only detected in QS samples with jujube powder addition, which indicated that jujube powder can enhance both the content and quantity of the aroma profile of QS. The reason was that jujube powder can provide rich characteristic aroma compounds to the aroma profile of QS, such as acetic acid and hexanoic acid (Song et al., 2020), and produce new aroma substances by Maillard reaction during the baking process (Qiao et al., 2023; Song et al., 2022). PCA was carried out further to classify different QS samples based on GC–MS data.

**TABLE 2:** Volatile compositions of QS with different additions of jujube powder (0–30%) analyzed by GC–MS.

Peak	Retention time	Compounds	Molecular structure	Content (μg/kg)						
				QS‐0	QS‐5	QS‐10	QS‐15	QS‐20	QS‐25	QS‐30
1	1.584	Acetaldehyde	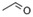	191.1 ± 7.28a	258.1 ± 9.15b	302.6 ± 9.81c	338.9 ± 14.0d	403.5 ± 10.7e	482.3 ± 9.16f	531.9 ± 16.7 g
2	1.959	2‐Methylpropanal	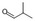	86.45 ± 2.31c	80.72 ± 2.08bc	78.37 ± 2.06b	77.26 ± 1.99b	75.40 ± 2.52b	73.17 ± 3.01ab	70.61 ± 1.79a
3	2.503	Ethyl acetate	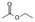	35.79 ± 1.52a	38.31 ± 2.06a	46.57 ± 1.86b	60.25 ± 1.9c1	61.18 ± 2.15c	62.03 ± 1.28c	62.55 ± 1.74c
4	2.812	Methylbutanal	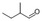	174.4 ± 6.67a	272.7 ± 12.7b	400.8 ± 14.4c	442.7 ± 10.9d	837.3 ± 22.5e	1205 ± 22.9f	1212 ± 19.5f
5	3.022	Ethanol	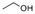	1126 ± 18.3a	1184 ± 25.0a	1990 ± 26.6b	2232 ± 52.7c	2536 ± 47.1d	3021 ± 55.3e	3581 ± 61.4f
6	3.758	2,3‐Butanedione	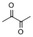	280.4 ± 8.78d	276.6 ± 10.7d	236.5 ± 6.81c	232.9 ± 5.58c	212.6 ± 7.11b	206.5 ± 7.06b	154.6 ± 3.54a
7	4.943	Ethyl butyrate	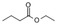	–	17.05 ± 1.23a	26.42 ± 1.09b	41.71 ± 2.26c	49.55 ± 1.96d	68.74 ± 2.63e	82.45 ± 2.88f
8	5.237	(E)‐2‐butenal	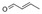	–	58.61 ± 1.42a	81.09 ± 2.21b	114.6 ± 3.90c	154.0 ± 3.81d	301.5 ± 5.38e	949.9 ± 17.1f
9	5.519	2,3‐Pentanedione	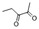	51.03 ± 1.57e	46.28 ± 1.36d	45.13 ± 1.09d	42.06 ± 1.14d	31.67 ± 1.37c	24.33 ± 0.66b	19.5 ± 0.72a
10	6.084	Hexanal	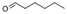	984.1 ± 12.6 g	827.5 ± 13.0f	739.8 ± 10.7e	644.1 ± 9.25d	482.5 ± 8.33c	296.6 ± 5.19b	125.6 ± 3.85a
11	6.126	2‐Methylpropanol	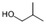	774.3 ± 8.72 g	728.3 ± 6.33f	607.3 ± 6.75e	373.8 ± 5.18d	310.8 ± 4.26c	283.7 ± 4.05b	251.4 ± 4.62a
12	9.383	3‐Methylbutanol	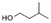	3487 ± 65.8e	3159 ± 81.3d	3096 ± 66.4d	2206 ± 39.1c	2013 ± 53.0c	1854 ± 47.8b	1525 ± 50.6a
13	10.991	Ethyl hexanoate	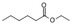	–	51.63 ± 3.02a	99.36 ± 4.74b	158.3 ± 4.18c	210.5 ± 6.65d	280.8 ± 6.19e	314.6 ± 6.77f
14	11.588	Acetic acid	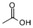	–	7.86 ± 0.33a	13.44 ± 0.57b	22.51 ± 0.49c	47.89 ± 1.42d	56.39 ± 1.75e	70.34 ± 1.19f
15	11.675	Acetoin	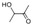	170.3 ± 6.14a	273.9 ± 5.92b	320.1 ± 3.37c	360.4 ± 5.22d	504.9 ± 7.04e	819.2 ± 7.76f	1099 ± 16.1 g
16	13.993	1‐Hexanol	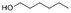	30.03 ± 0.47a	49.38 ± 0.29b	58.26 ± 0.86c	78.96 ± 1.26d	105.7 ± 1.97e	134.9 ± 2.22f	156.0 ± 1.98 g
17	14.995	Dipropyl disulfide	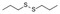	58.06 ± 0.36a	197.2 ± 4.20b	348.3 ± 3.74c	488.6 ± 6.03d	626.0 ± 10.5e	803.9 ± 11.2f	952.2 ± 12.4 g
18	16.999	Furfural	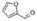	–	13.64 ± 0.52a	25.18 ± 0.48b	37.09 ± 0.71c	53.99 ± 1.33d	88.72 ± 1.66e	137.9 ± 3.39f
19	17.410	Ethyl octanoate	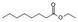	32.25 ± 0.34a	53.45 ± 0.18b	53.97 ± 0.44b	54.27 ± 0.36b	55.96 ± 0.62b	56.05 ± 0.77b	56.59 ± 0.56b
20	18.139	Hexanoic acid	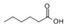	–	20.07 ± 0.45a	38.63 ± 0.43b	52.10 ± 0.82c	66.38 ± 0.77d	81.42 ± 0.74e	96.89 ± 1.89f
21	18.661	Benzaldehyde	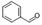	–	88.05 ± 1.15a	185.4 ± 1.78b	255.7 ± 4.24c	386.9 ± 5.16d	526.6 ± 7.06e	627.2 ± 6.67f
22	18.775	2,3‐Butanediol	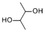	–	185.6 ± 10.4a	223.7 ± 8.54b	362.2 ± 9.71c	443.7 ± 11.2d	572.9 ± 16.0e	704.3 ± 12.5f
23	21.665	Benzeneacetaldehyde	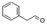	–	23.06 ± 0.88a	89.75 ± 2.01b	160.3 ± 4.71c	272.9 ± 3.83d	351.2 ± 6.67e	478.1 ± 5.36f
24	22.410	Ethyl benzoate	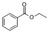	–	8.53 ± 0.61a	14.66 ± 0.39b	23.74 ± 0.71c	37.57 ± 0.55d	60.61 ± 1.32e	82.99 ± 3.02f
25	27.647	Phenylethyl alcohol	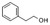	–	154.3 ± 4.62a	191.4 ± 5.12b	226.1 ± 4.48c	288.3 ± 6.06d	345.3 ± 12.1e	398.8 ± 9.78f
26	34.784	Indole	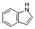	–	14.25 ± 0.62a	15.06 ± 0.39ab	16.55 ± 0.55bc	16.73 ± 0.62bc	16.91 ± 0.57c	16.90 ± 0.49c

*Note*: QS‐0, QS‐5, QS‐10, QS‐15, QS‐20, QS‐25, and QS‐30 was quinoa snack with the addition of jujube powder of 0%, 5%, 10%, 15%, 20%, 25%, and 30%, respectively. Data are represented as the mean ± SD (standard deviation). Mean values with different lower case letters in the same column correspond to significant differences at *p* < .05.

#### 3.3.2 PCA analysis

**FIGURE 3:**
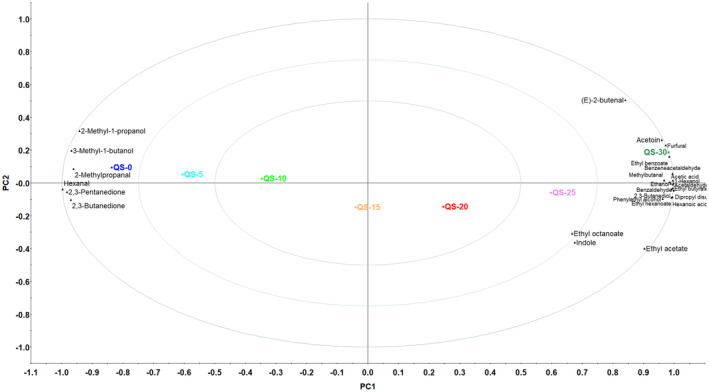
Principal component analysis (PCA) of quinoa snack (QS) samples based on the data from GC–MS.

In order to make clear the characteristics of the volatile profiles of QS samples with different additions of jujube powders, PCA was also applied based on the GC–MS data (Table 2). The PCA chart of different QS samples was presented in Figure 3, and it clearly showed QS samples were well separated, indicating the characteristic volatile compounds of QS samples with different additions of jujube powders were significantly different (Song, Shao, et al., 2021; Song, Yan, et al., 2021). As shown in Figure 3, QS‐0, QS‐5, QS‐10, QS‐15, QS‐20, QS‐25, and QS‐30 were separated one by one along the x‐axis, which indicated that with the increasing addition of jujube powders, the volatile profile of the QS sample was significant. Moreover, QS‐0 was closest to 2‐methylproanal, hexanal, 2,3‐pentanedione, 2,3‐butanedione, 3‐methylbutanol, and 2‐methylpropanol, which indicated that QS‐0 could be characterized by these aroma compositions because these volatile compounds showed the highest content in QS‐0, respectively (Table 2). For the highest content, QS‐30 could be characterized by furfural, acetoin, ethyl benzoate, benzeneacetaldehyde, methylbutanal, acetic acid, 1‐hexanol, benzaldehyde, 2,3‐butanediol, hexanoic acid, and ethyl hexanoate (Figure 3). Generally, PCA was effective in distinguishing the aroma profiles of different QS samples.

### 3.4 Combination analysis of e‐nose and GC–MS

In terms of e‐nose, W1S (sensitive to methane) was the most significant in QS samples (Figure 1b). It was mainly caused by the aroma compounds of 3‐methylbutanol, 2‐methylpropanol, 2‐methylpropanal, and methylbutanal (methyl group, –CH_3_), and 3‐methylbutanol (from 1525 μg/kg in QS‐30 to 3487 μg/kg in QS‐0) was the most common in QS samples. The response of W2S (sensitive to alcohols) of e‐nose to QS samples was also significant, which was because of ethanol, 2‐methylpropanol, 3‐methylbutanol, 1‐hexanol, 2,3‐butanediol, and phenylethyl alcohol (hydroxyl group, –OH) in QS samples, and the strongest response of W2S was to QS‐30 for its highest content of ethanol (3581 μg/kg), 1‐hexanol (156.0 μg/kg), 2,3‐butanediol (704.3 μg/kg), and phenylethyl alcohol (398.8 μg/kg; Table 2). Considering the PCA results of e‐nose (Figure 2) and GC–MS (Figure 3), both of them could well distinguish the volatile profile differences of QS samples with different additions of jujube powders and showed similar graphical layout, and the QS‐30 with the highest jujube content (30%) presented the strongest aroma profile. Generally, the e‐nose results (Figures 1 and 2) were well in agreement with the GC–MS data (Table 1, Figure 3), and their combination could be more effective in the volatile profile analysis of QS samples.

## 4 CONCLUSION

A quinoa snack (QS) with different jujube additions (from 5% to 30%) was successfully developed, and a total of 26 aroma compounds were identified in QS samples by GC–MS. 3‐Methylbutanol, ethanol, hexanal, 2‐methylpropanol, 2,3‐butanedione, methylbutanal, acetaldehyde, and acetoin were common in QS samples. The aroma profile of the QS sample was significantly (*p* < .05) enhanced by the addition of jujube powder, and the aroma profile differences of the QS samples could be well distinguished by PCA. In conclusion, QS‐30 with the highest jujube content (30%) presented the strongest aroma profile, which had potential for market applications.

## AUTHOR CONTRIBUTIONS

**Jianxin Song:** Project administration (lead); supervision (lead); writing – original draft (lead). **Jiayi Liu:** Methodology (lead). **Kaile Wang:** Data curation (lead). **Lei Gao:** Investigation (lead). **Xiaodong Wang:** Project administration (equal); supervision (equal). **Jian Peng:** Supervision (lead). **Ning Wang:** Data curation (equal).

## CONFLICT OF INTEREST STATEMENT

All the authors declare that they have no conflict of interest.

## Data Availability

The data that support the findings of this study are available on request from the corresponding author. The data are not publicly available due to privacy or ethical restrictions.
